# Time to Recovery of Feeding After Alveolar Bone Graft Is Associated With Postoperative Nausea and Vomiting Within 2 Hours in Children

**DOI:** 10.1177/1179556519855387

**Published:** 2019-06-14

**Authors:** Kaoru Yamashita, Toshiro Kibe, Sachi Ohno, Minako Uchino, Yurina Higa, Ayako Niiro, Norifumi Nakamura, Mitsutaka Sugimura

**Affiliations:** 1 Department of Dental Anesthesiology, Field of Oral and Maxillofacial Rehabilitation, Developmental Therapeutics Course, Graduate School of Medical and Dental Sciences, Kagoshima University, Kagoshima, Japan; 2 Department of Oral and Maxillofacial Surgery, Field of Oral and Maxillofacial Rehabilitation, Developmental Therapeutics Course, Graduate School of Medical and Dental Sciences, Kagoshima University, Kagoshima, Japan

**Keywords:** alveolar bone graft, children, postoperative nausea and vomiting, time to recovery after feeding

## Abstract

**Objective:**

To examine the relationship between the method of anesthesia for alveolar bone graft surgery and postoperative nausea and vomiting (PONV) based on the difference in surgical timing and to assess factors related to the postoperative quality of life.

**Design:**

Retrospective observational study.

**Setting:**

Hospital.

**Participants:**

Patients with cleft lip and palate who underwent alveolar bone graft surgery under general anesthesia. The subjects were divided into two groups based on surgical timing: secondary bone graft (SBG) and late secondary bone graft (LSBG) groups.

**Main Outcome Measures:**

Relationship between time to recovery of feeding and the types of anesthesia, PONV, and postoperative pain period.

**Results:**

The mean patient age was 9.97 ± 1.33 years in the SBG group and 15.39 ± 0.31 years in the LSBG group. In the SBG group, patients who were administered fentanyl or remifentanil had significantly higher incidence of PONV than those who were not administered these drugs. In the SBG group, the time to recovery of feeding was significantly longer in patients experiencing PONV within 2 hours or that lasted for 24 hours than in those without PONV. In the LSBG group, there was no significant difference regarding any of the above factors.

**Conclusions:**

Our results suggest that the occurrence of PONV within 2 hours or lasting for 24 hours postoperatively in school-age children prolonged the time to recovery of feeding. This indicates that the time to recovery of feeding can be predicted based on the occurrence of PONV within the first 2 hours.

## Introduction

Cleft lip and palate is a congenital disorder that requires multiple surgeries. Alveolar bone grafting is performed to reconstruct the alveolar cleft to allow eruption of normal dentition in patients with alveolar cleft.^1^ The surgical timing depends on the condition of the patient’s dentition. If performed before canine tooth eruption, it is called secondary bone graft (SBG) and if performed after the eruption of permanent teeth, it is called late secondary bone graft (LSBG).^2,3^ The surgery is the same; however, the age of the patient is different in the two surgeries as the surgical timing is different in both. Therefore, anesthetic management varies depending on the age.

Depending on the age of the patient, the type of anesthetic used and the amount of narcotics are also different. Anesthetics and narcotics are risk factors for postoperative nausea and vomiting (PONV), and it has been seen that differences in anesthesia management among children and adolescents may also cause differences in the occurrence of PONV.^4–6^ PONV significantly decreases the patient’s quality of life (QOL).^7–9^ Vomiting may cause dehydration, fever, and electrolyte disturbances. It is often difficult to treat the original cause^10;^ therefore, counter measures against PONV are extremely important. In addition, some patients have poor oral intake for several days after surgery^11;^ therefore, it is important to evaluate the delay in oral intake. Hence, we felt that it was important to evaluate the prolongation of recovery of feeding and manage the patient’s feeding at an early stage. There have been studies on PONV in children, but the studies include PONV after different surgeries.^7,12–14^ There have been no previous reports comparing the occurrence of PONV in children undergoing the same surgery in two different surgical timings. Therefore, in this study, we aimed to examine the relationship between the type of anesthesia for alveolar bone graft surgeries and PONV based on the difference in surgical timing and to study the factors related to postoperative QOL.

## Methods

This retrospective study was approved by the Clinical Ethics Committee of our hospital, which waived the requirement of informed consent because of the retrospective nature of the study; however, patients were informed of the possible use of data for research. This study included 90 patients with alveolar cleft who underwent alveolar bone graft surgery under general anesthesia from March 2012 to December 2016. We compiled information using anesthesia records, surgical records, and electronic charts. We excluded patients whose information was missing in the charts. Subjects were divided into two groups, the SBG group and the LSBG group, based on surgical timing. All surgeries were performed by the one experienced alveolar bone graft team using the technique described by Boyne and Sands.^15^ Surgeries of donor site were performed using the technique described by Robertson and Barron.^16^ The age, sex, height, weight, alveolar cleft type, operation time, anesthesia time, amount of blood loss, anesthesia method, presence or absence of nausea and vomiting after surgery, postoperative pain period, and time till oral intake was resumed were investigated. The following examination parameters related to postoperative QOL were investigated: PONV (within 2 hours, lasting for 24 hours), postoperative pain period, time to recovery of feeding (number of days until children and adolescents could eat more than 50% of their meal), and the anesthetic method used for surgery.

For statistical analysis, the chi-square test, the Mann–Whitney *U* test, and logistic regression analysis were performed, and *P* < .05 was regarded as statistically significant. Statistical analysis was performed using GraphPad Prism, version 6 (San Diego, CA).

## Results

### Patient characteristics in the SBG and LSBG groups, PONV, and time to recovery of feeding

There were 68 patients in the SBG group with a mean age of 9.97 ± 1.33 years, mean body weight of 30.46 ± 6.34 kg, and mean height of 134.79 ± 7.78 cm (Table 1). The LSBG group consisted of 22 patients with a mean age of 15.39 ± 0.31 years, mean body weight of 46.09 ± 7.34 kg, and mean height of 157.95 ± 9.2 cm. Surgical time and anesthesia time were longer in the SBG group. In terms of the cleft type (Table 1), the proportion of unilateral cleft lip and alveolus was higher in the SBG group. A significantly larger number of patients in the LSBG group underwent rapid induction. In both groups, the anesthetic selected most often was sevoflurane; and a significantly greater number of patients in the SBG group received sevoflurane compared to other anesthetic agents.

**Table 1.** Patient characteristics in the secondary bone graft and late secondary bone graft groups, postoperative nausea and vomiting, and time to recovery of feeding.

	SBG (*n* = 68)	LSBG (*n* = 22)	Total (*n* = 90)	*P*
Age (years)	9.97 ± 1.33	15.39 ± 0.31	11.29 ± 2.71	<.01
Weight (kg)	30.46 ± 6.34	46.09 ± 7.34	34.28 ± 9.41	<.01
Height (cm)	134.79 ± 7.78	157.95 ± 9.207	140.45 ± 12.87	<.01
Male/female	33/35	13/9	46/44	.38
Operation time (min)	162.29 ± 65.92	208.18 ± 88.21	173.51 ± 74.17	<.01
Anesthesia time (min)	252.04 ± 74.43	305.18 ± 88.53	265.03 ± 8.52	<.01
Bleeding (min)	82 ± 72.82	106.04 ± 71.47	87.87 ± 72.83	.02
Cleft type				
Unilateral cleft lip and alveolus	27	2	29	.048
Unilateral cleft lip and palate	25	11	36	
Bilateral cleft lip and alveolus	2	2	4	
Bilateral cleft lip and palate	14	7	21	
Induction				
Slow	8	0	8	<.01
Rapid (Isozol)	40	4	44	
Rapid (propofol)	20	18	38	
Anesthetics and narcotics				
Sevoflurane	60	14	74	<.01
Propofol	8	8	16	
None	22	9	31	.46
N_2_O	46	13	59	
None	60	18	78	.44
Fentanyl	8	4	12	
None	21	3	24	.11
Remifentanil	47	19	66	
Postoperative analgesic				
Acetaminophen	39	12	51	.20
Flurbiprofen axetil	14	8	22	
PONV				
PONV 24 h	8	0	8	<.01
PONV 0-2 h	14	2	16	.18
Postoperative pain period (day)	2.16 ± 0.94	1.54 ± 0.86	2.01 ± 0.95	.01
Time to recovery of feeding (day)	3.25 ± 2.2	2.36 ± 1.29	3.03 ± 2.05	.12

Abbreviations: LSBG, late secondary bone graft; PONV, postoperative nausea and vomiting; SBG, secondary bone graft.

In addition, the use of acetaminophen for postoperative analgesia was higher in the SBG group, while flurbiprofen axetil was used in both the SBG and LSBG groups. In the SBG group, the postoperative pain period was significantly longer, the incidence of PONV within the first 2 hours was higher, and the time to recovery of feeding was longer, compared to the LSBG group.

### Difference in the time to recovery of feeding depending on the presence or absence of PONV within 2 hours and lasting for 24 hours in the SBG and LSBG groups

In the SBG group, the time to recovery of feeding in patients without PONV that lasted for 24 hours was 3 ± 0. 276 days, and the time to recovery of feeding in patients with PONV that lasted for 24 hours was 5.12 ± 0.66 days. These results suggest that the time to recovery of feeding in patients without PONV is longer than in those with PONV. In the LSBG group, none of the patients had PONV; therefore, the time to recovery of feeding could not be evaluated.

**Figure 1.**
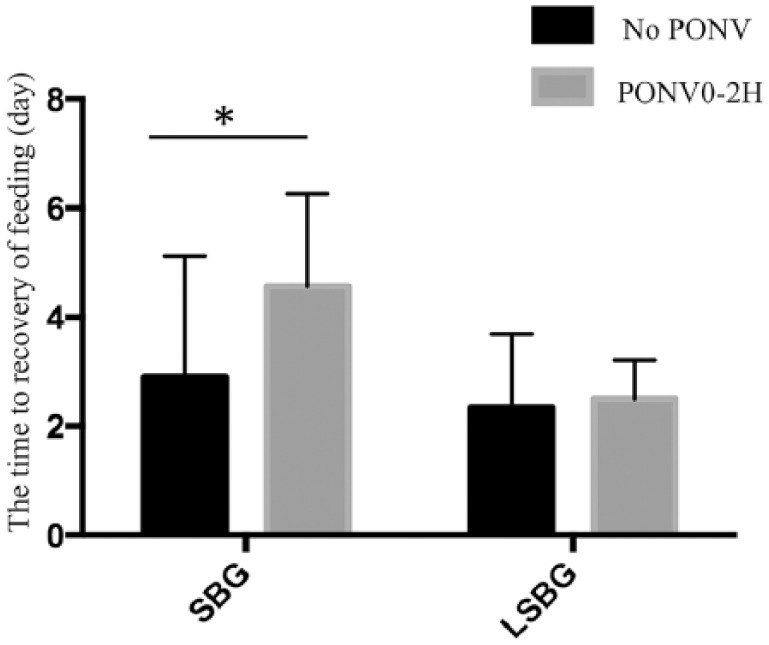
Difference in the time to recovery of feeding depending on the presence or absence of postoperative nausea and vomiting within 2 hours in the secondary bone graft and late secondary bone graft groups. Abbreviations: LSBG, late secondary bone graft; PONV, postoperative nausea and vomiting; SBG, secondary bone graft.

In the SBG group, the time to recovery of feeding in patients with PONV within 2 hours was 4.57 ± 1.69 days; this was significantly longer compared to that (2.9 ± 2.2 days) in patients without PONV within 2 hours. On the contrary, no significant difference was observed in the LSBG group (Figure 1).

### Univariate analysis of anesthesia-related factors as risk factors for occurrence of PONV within 2 hours in the SBG and LSBG groups

In the SBG group, the occurrence of PONV within 2 hours was significantly higher in cases where nitrous oxide, fentanyl, and remifentanil were used compared to cases where these agents were not used (Table 2).

**Table 2.** Univariate analysis of anesthesia-related factors as risk factors for the occurrence of postoperative nausea and vomiting within 2 hours in the secondary bone graft and late secondary bone graft groups.

SBG	No PONV (*n* = 54)	PONV 0-2 hours (*n* = 14)	*P*
Age (years)	9.9 ± 1.27	10.21 ± 1.57	.6
Weight (kg)	30.5 ± 5.94	32.03 ± 7.72	.27
Height (cm)	134.4 ± 8.45	136.26 ± 5.63	.26
Male/female	29/25	25/5	.47
Operation time (min)	103.11 ± 68.54	159.14 ± 54.79	.84
Anesthesia time (min)	256.72 ± 79.86	234 ± 45.86	.37
Bleeding (min)	78.7 ± 61.3	94.42 ± 108.4	.93
Cleft type			
Unilateral cleft lip and alveolus	24	3	.33
Unilateral cleft lip and palate	18	7	
Bilateral cleft lip and alveolus	2	0	
Bilateral cleft lip and palate	10	4	
Induction			
Slow	7	1	.81
Rapid (Isozol)	31	9	
Rapid (Propofol)	16	4	
Anesthetics and narcotics			
Sevoflurane	46	14	.13
Propofol	8	0	
None	21	1	.023
N_2_O	33	13	
None	50	10	.028
Fentanyl	4	4	
None	20	1	.03
Remifentanil	34	13	
Postoperative analgesic			
Acetaminophen	27	12	.07
Flurbiprofen axetil	13	1	
Postoperative recovery			
Postoperative pain period (days)	2.18 ± 1.01	2.07 ± 0.61	.49
Time to recovery of feeding (days)	2.9 ± 2.2	4.57 ± 1.69	<.01
LSBG	No PONV (*n* = 20)	PONV 0-2 hours (*n* = 2)	*P*
Age (years)	15.56 ± 1.38	13.7 ± 2.06	.17
Weight (kg)	45.99 ± 6.43	47.1 ± 18.52	.9
Height (cm)	150.58 ± 9.13	151.75 ± 10.53	.36
Male/Female	8/0	12/0	.26
Operation time (min)	209.5 ± 92.55	195 ± 16.97	.64
Anesthesia time (min)	306.3 ± 92.85	294 ± 22.62	.56
Bleeding (min)	110.15 ± 73.77	65 ± 12.72	.2
Cleft type			
Unilateral cleft lip and alveolus	1	0	.33
Unilateral cleft lip and palate	11	1	
Bilateral cleft lip and alveolus	2	0	
Bilateral cleft lip and palate	6	1	
Induction			
Slow	0	0	.22
Rapid (Isozol)	3	1	
Rapid (propofol)	17	1	
Anesthetics and narcotics			
Sevoflurane	12	2	.26
Propofol	8	0	
None	9	0	.21
N_2_O	11	2	
None	16	2	.48
Fentanyl	4	0	
None	3	0	.55
Remifentanil	17	2	
Postoperative analgesic			
Acetaminophen	11	1	.25
Flurbiprofen axetil	7	1	
Postoperative recovery			
Postoperative pain period (day)	1.5 ± 0.88	2	.42
Time to recovery of feeding (day)	2.35 ± 1.34	2.5 ± 0.7	.86

Abbreviations: PONV, postoperative nausea and vomiting; LSBG, late secondary bone graft; SBG, secondary bone graft.

In the LSBG group, no significant differences were observed in terms of the incidence of PONV with reference to anesthesia-related factors (Table 2).

### Multivariate analysis of anesthesia-related factors as risk factors for the occurrence of PONV within 2 hours in the SBG group

In the SBG group, the occurrence of PONV within 2 hours was related to the use of fentanyl and remifentanil (Table 3).

**Table 3.** Multivariate analysis of anesthesia-related factors as risk factors for the occurrence of postoperative nausea and vomiting within 2 hours in the secondary bone graft group.

SBG	No PONV	PONV 0-2 hours	Total	*P*
None	21	1	22	n.s.
N_2_O	33	13	46	
None	50	10	60	.035
Fentanyl	4	4	8	
None	20	1	21	.035
Remifentanil	34	13	47	

Abbreviations: n.s., not significant; PONV, postoperative nausea and vomiting; SBG, secondary bone graft.

## Discussion

Many patients complain that PONV is more difficult to endure than postoperative pain, which reduces patient satisfaction. Furthermore, it has been shown that persistence of PONV causes surgical wound dehiscence, postoperative bleeding, aspiration pneumonia, and other postoperative complications.^17–19^ In addition, delays in recovery of feeding are also considered to cause delay in postoperative recovery.

In terms of the age group of the patients, the SBG group consisted of school-age children, and the LSBG group consisted of adolescents. Therefore, there were differences in the heights and weights. A significant difference was also observed in the alveolar cleft type. The aforementioned results can be attributed to the fact that congenital defects of the permanent teeth in the cleft lip and alveolus are less common than those of the cleft lip and palate, and the surgical timing in the SBG group is earlier.^20,21^ With regard to patient age, slow induction is selected for school-age children when injection is difficult because of patient uncooperativeness. On the contrary, propofol is often used for maintenance of anesthesia for adolescent patients. Moreover, PONV in school-age children was increased due to the administration of narcotics; this result is in agreement with past reports.^9,22,23^ In the SBG group, patients who were administered narcotics had a significantly higher incidence of PONV within the first 2 hours compared to patients in whom narcotics was not administered. However, no significant difference was observed in this respect in the LSBG group. The use of opioids during and after surgery is a risk factor for PONV.^4^ Fentanyl citrate indirectly activates the dopamine receptor of the chemoreceptor trigger zone by binding to the medullary μ opioid receptor and causes PONV.^22^ Therefore, analgesics other than fentanyl might be better in SBG patients. It has been reported that the use of remifentanil (which is short acting) decreases PONV compared to fentanyl citrate^24^; however, in our study, there was no decrease in PONV with remifentanil.

Acetaminophen and flurbiprofen axetil are used for postoperative analgesia besides fentanyl. However, our study did not evaluate the postoperative pain period; therefore, we could not assess the effect of pain on PONV.

However, in the SBG group, the time to recovery of feeding was significantly longer in patients in whom the PONV lasted for 24 hours than patients without PONV. Furthermore, in the SBG group, the time to recovery of feeding in patients with PONV within the first 2 hours was significantly longer than in patients without PONV. On the contrary, no significant difference was observed in the LSBG group. The lack of a statistically significant difference between PONV and the time to recovery of feeding was probably because of the small sample size in the LSBG group.

In other words, it can be concluded that PONV within 2 hours and lasting for 24 hours after surgery in school-age children is involved in prolonging the time to recovery of feeding. In order to shorten the time to recovery of feeding, it is necessary to reduce the occurrence of PONV. The incidence of PONV is related to the use of narcotics. Therefore, reducing the use of narcotics may be necessary to shorten the time to recovery of feeding.

There have been previous studies on PONV in children that have compared and examined the condition separately for each age group. However, there have not been many reports that have given consideration to patients undergoing the same type of surgery.^12–14^ In this study, patients who underwent the same surgery could be compared for different age groups due to differences in surgical timing. Therefore, we believe that new knowledge on PONV in children and adolescents was obtained from this study.

All patients undergo lip repair or lip and palate repair by 2 years of age. Therefore, patients who undergo alveolar bone grafting have no memory of the primary surgery. Alveolar bone grafting is the first surgery that remains memorable for patients with cleft lip and palate. Therefore, it is unlikely that memory of the previous surgery would cause PONV.

Our study has some limitations. Since the number of days until discharge is predetermined in our facility, in this study, it was not possible to evaluate the time required for postoperative recovery based on the number of days until discharge. Another limitation of the study was that the number of cases in the LSBG group was not sufficient. This was because in order to align conditions, such as the surgical method and the anesthesia method, it was necessary to limit the study duration to this period.

## Conclusion

Occurrence of PONV within 2 hours and lasting for 24 hours after surgery in school-age children prolonged the time to recovery of feeding. We believe that the time to recovery of feeding can be predicted based on the occurrence of PONV within 2 hours.

## References

[1] Bergland O Semb G Abyholm FE. Elimination of the residual alveolar cleft by secondary bone grafting and subsequent orthodontic treatment. Cleft Palate J. 1986;23:175–205. 3524905

[2] Kazemi A Stearns JW Fonseca RJ. Secondary grafting in the alveolar cleft patient. Oral Maxillofac Surg Clin North Am. 2002;14:477–490. 18088649 10.1016/s1042-3699(02)00042-0

[3] Elhaddaoui R Bahije L Zaoui F Rerhrhaye W. Timing of alveolar bone graft and sequences of canine eruption in cases of cleft lip and palate: a systematic review. Orthod Fr. 2017;88:193–198. 28597839 10.1051/orthodfr/2017011

[4] Apfel CC Korttila K Abdalla M et al A factorial trial of six interventions for the prevention of postoperative nausea and vomiting. N Engl J Med. 2004;350:2441–2451. 15190136 10.1056/NEJMoa032196 PMC1307533

[5] Bordes M Cros AM. Inhalation induction with sevoflurane in paediatrics: what is new? Ann Fr Anesth Reanim. 2006;25:413–416. 16455225 10.1016/j.annfar.2005.10.017

[6] Rawlinson A Kitchingham N Hart C McMahon G Ong SL Khanna A. Mechanisms of reducing postoperative pain, nausea and vomiting: a systematic review of current techniques. Evid Based Med. 2012;17:75–80. 22419772 10.1136/ebmed-2011-100265

[7] Rose JB Watcha MF. Postoperative nausea and vomiting in paediatric patients. Br J Anaesth. 1999;83:104–117. 10616338 10.1093/bja/83.1.104

[8] Alsaif A Alqahtani S Alanazi F Alrashed F Almutairi A. Patient satisfaction and experience with anesthesia: a multicenter survey in Saudi population. Saudi J Anaesth. 2018;12:304–310. 29628845 10.4103/sja.SJA_656_17 PMC5875223

[9] Kocaturk O Keles S Omurlu IK. Risk factors for postoperative nausea and vomiting in pediatric patients undergoing ambulatory dental treatment. Niger J Clin Pract. 2018;21:597–602. 29735860 10.4103/njcp.njcp_129_17

[10] Singhi SC Shah R Bansal A Jayashree M. Management of a child with vomiting. Indian J Pediatr. 2013;80:318–325. 23340985 10.1007/s12098-012-0959-6

[11] Chauvin C Schalber-Geyer AS Lefebvre F et al Early postoperative oral fluid intake in paediatric day case surgery influences the need for opioids and postoperative vomiting: a controlled randomized trialdagger. Br J Anaesth. 2017;118:407–414. 28203729 10.1093/bja/aew463

[12] Cohen MM Cameron CB Duncan PG. Pediatric anesthesia morbidity and mortality in the perioperative period. Anesth Analg. 1990;70:160–167. 2301747 10.1213/00000539-199002000-00005

[13] Karlsson E Larsson LE Nilsson K. Postanaesthetic nausea in children. Acta Anaesthesiol Scand. 1990;34:515–518. 1978765 10.1111/j.1399-6576.1990.tb03136.x

[14] Lerman J. Surgical and patient factors involved in postoperative nausea and vomiting. Br J Anaesth. 1992;69:24S-32S. 1486011 10.1093/bja/69.supplement_1.24s

[15] Boyne PJ Sands NR. Secondary bone grafting of residual alveolar and palatal clefts. J Oral Surg. 1972;30:87–92. 4550446

[16] Robertson IM Barron JN. A method of treatment of chronic infective osteitis. J Bone Joint Surg Am. 1946;28:19–28. 21008069

[17] Macario A Weinger M Truong P Lee M. Which clinical anesthesia outcomes are both common and important to avoid? The perspective of a panel of expert anesthesiologists. Anesth Analg. 1999;88:1085–1091. 10320175 10.1097/00000539-199905000-00023

[18] Apfel CC Kranke P Katz MH et al Volatile anaesthetics may be the main cause of early but not delayed postoperative vomiting: a randomized controlled trial of factorial design. Br J Anaesth. 2002;88:659–668. 12067003 10.1093/bja/88.5.659

[19] Scuderi PE Conlay LA. Postoperative nausea and vomiting and outcome. Int Anesthesiol Clin. 2003;41:165–174. 14574220 10.1097/00004311-200341040-00012

[20] Brattstrom V McWilliam J. The influence of bone grafting age on dental abnormalities and alveolar bone height in patients with unilateral cleft lip and palate. Eur J Orthod. 1989;11:351–358. 2687012 10.1093/oxfordjournals.ejo.a036006

[21] Suzuki A Watanabe M Nakano M Takahama Y. Maxillary lateral incisors of subjects with cleft lip and/or palate: part 2. Cleft Palate Craniofac J. 1992;29:380–384. 1643071 10.1597/1545-1569_1992_029_0380_mliosw_2.3.co_2

[22] Apfel CC Heidrich FM Jukar-Rao S et al Evidence-based analysis of risk factors for postoperative nausea and vomiting. Br J Anaesth. 2012;109:742–753. 23035051 10.1093/bja/aes276

[23] Gan TJ Diemunsch P Habib AS et al Consensus guidelines for the management of postoperative nausea and vomiting. Anesth Analg. 2014;118:85–113. 24356162 10.1213/ANE.0000000000000002

[24] Rama-Maceiras P Ferreira TA Molins N Sanduende Y Bautista AP Rey T. Less postoperative nausea and vomiting after propofol + remifentanil versus propofol + fentanyl anaesthesia during plastic surgery. Acta Anaesthesiol Scand. 2005;49:305–311. 15752393 10.1111/j.1399-6576.2005.00650.x

